# Evaluation of Comparative Surveillance Strategies of Circulating Tumor DNA, Imaging, and Carcinoembryonic Antigen Levels in Patients With Resected Colorectal Cancer

**DOI:** 10.1001/jamanetworkopen.2022.1093

**Published:** 2022-03-08

**Authors:** Marwan Fakih, Jaideep Sandhu, Chongkai Wang, Jae Kim, Yi-Jen Chen, Lily Lai, Kurt Melstrom, Andreas Kaiser

**Affiliations:** 1 Department of Medical Oncology and Therapeutics Research, City of Hope Comprehensive Cancer Center, Duarte, California; 2 Department of Surgery, City of Hope National Medical Center, Duarte, California; 3 Department of Radiation Oncology, City of Hope National Medical Center, Duarte, California

## Abstract

**Importance:**

A circulating tumor DNA (ctDNA) assay (Signatera; Natera) has been marketed for use in the surveillance of resected colorectal cancer despite limited data supporting such practice.

**Objective:**

To compare a ctDNA assay with standard radiographic imaging and measurement of carcinoembryonic antigen (CEA) levels, per National Comprehensive Cancer Network guidelines, in the surveillance of resected colorectal cancer.

**Design, Setting, and Participants:**

This retrospective, single-center cohort study evaluated surveillance strategies of ctDNA, imaging, and measurement of CEA levels in patients with resected colorectal cancer from September 1, 2019, to November 30, 2021.

**Main Outcomes and Measures:**

The sensitivity and specificity of ctDNA, imaging, measurement of CEA levels, and combination of imaging plus measurement of CEA levels in detecting a confirmed recurrence of colorectal disease. A confirmed recurrence was defined as a positive ctDNA finding or a finding on imaging confirmed by biopsy, CEA level elevation, or subsequent tumor radiographic dynamics.

**Results:**

A total of 48 patients with curatively resected colorectal cancer satisfied the inclusion criteria for this study (28 men [58.3%]; median age, 60 [IQR, 34-85] years) and underwent surveillance by ctDNA, imaging, and measurement of CEA levels. Fifteen patients had disease recurrence during surveillance. Positive ctDNA findings confirmed disease recurrence in 8 patients; imaging, in 9 patients; CEA levels, in 3 patients; and combined imaging plus CEA levels, in 11 patients. Numerically, ctDNA did not perform better than imaging in detecting recurrence, with sensitivities of 53.3% (95% CI, 27.4%-77.7%) and 60.0% (95% CI, 32.9%-82.5%), respectively (*P* > .99). The combination of imaging plus measurement of CEA levels (sensitivity, 73.3% [95% CI, 44.8%-91.1%]) had a numerical advantage compared with ctDNA in identifying recurrence (*P* = .55). In addition, no significant difference was noted among ctDNA (median, 14.3 months), imaging (median, 15.0 months), or imaging plus measurement of CEA levels (median, 15.0 months) in the time to identify disease recurrence.

**Conclusions and Relevance:**

The findings of this cohort study suggest that ctDNA assay may not provide advantages as a surveillance strategy compared with standard imaging combined with CEA levels when performed per National Comprehensive Cancer Network guidelines.

## Key Points

**Question:**

Is serial analysis of circulating tumor DNA (ctDNA) associated with improved sensitivity and earlier detection of recurrence compared with standard imaging and evaluation of carcinoembryonic antigen (CEA) levels per National Comprehensive Cancer Network guidelines in patients with resected colorectal cancer?

**Findings:**

In this cohort study of 48 patients with resected colorectal cancer, 15 had confirmed disease recurrence by imaging, of whom only 8 had a concurrent positive ctDNA finding. The combination of imaging and CEA measurement had better sensitivity compared with ctDNA in identifying disease recurrence (73.3% vs 53.3%).

**Meaning:**

The findings of this cohort study suggest that ctDNA assay provides no definitive advantage compared with standard imaging and CEA measurement in the surveillance of patients with resected colorectal cancer.

## Teaser

This cohort study compares the sensitivity and specificity of a circulating tumor DNA assay with imaging, measurement of carcinoembryonic antigen (CEA) levels, and imaging plus CEA measurement for surveillance in patients with resected colorectal cancer as recommended by the National Comprehensive Cancer Network guidelines.

## Introduction

Surveillance strategies in the management of resected locoregional and metastatic colorectal cancer have been investigated thoroughly during the last several decades.^1,2,3,4,5^ Although carcinoembryonic antigen (CEA) levels and intensive imaging have been investigated as sole or combination strategies in the surveillance of resected stage I to III disease, considerable disagreement remains regarding the impact of these assays on colorectal cancer outcome.^4,6,7^ Despite the limited benefits of measuring CEA levels and imaging on the overall survival among patients with colorectal cancer, these tests are still believed to have value in the early detection of metastatic disease recurrence, which can lead to curative surgery.^8^ The National Comprehensive Cancer Network (NCCN) currently recommends the surveillance of stage II and III colorectal cancer with measurement of CEA levels every 3 to 6 months for 2 years followed by every 6 months for 3 years. In addition, patients with stage II to III colorectal cancer are to undergo computed tomography (CT) of the chest, abdomen, and pelvis every 6 to 12 months for 2 years followed by yearly imaging for 3 years.^9^ For resected stage IV disease, the NCCN recommends a similar CEA surveillance strategy and intensive imaging, with a CT scan of the chest, abdomen, and pelvis every 3 to 6 months for 2 years followed by every 6 to 12 months for another 3 years.^9^ The European Society of Medical Oncology (ESMO) has largely endorsed a similar surveillance strategy.^10^ Neither the NCCN nor the ESMO recommend the use of circulating tumor DNA (ctDNA) assays for surveillance of colorectal cancer.

More recently, several studies have evaluated ctDNA as a surveillance strategy for resected colorectal cancer. Signatera (Natera) is a personalized, tumor-informed, multiplex polymerase chain reaction–based next-generation sequencing assay for ctDNA detection.^11^ Reveal (Guardant Health, Inc) is a tumor agnostic assay that simultaneously evaluates genomic mutations and methylations to detect residual disease and colorectal disease recurrence.^12^ Both assays are commercially available in the US. Many clinicians have elected to incorporate these ctDNA-based assays in the surveillance of resected colorectal cancer, despite limited supportive clinical data.^11,12,13,14,15^ The enthusiasm around these assays, particularly Signatera, was generated by a large observational surveillance trial^11^ that evaluated CEA levels, CT imaging, and ctDNA in patients with stage I to III colorectal cancer. In that study, patients underwent postoperative surveillance by ctDNA assay (Signatera) after adjuvant therapy and every 3 months for 36 months (along with measurement of CEA levels) and by radiographic imaging at 1 and 3 years as per the Danish Colorectal Cancer Group guidelines. This study showed that ctDNA assay identified disease recurrence at a median of 8.7 months before radiographic recurrence.^11^ The relevance of such clinical findings should be taken in the context of the surveillance frequency of imaging studies. The Danish Colorectal Cancer Group guidelines for CT surveillance frequency are considered substandard in the US by the American Society of Clinical Oncology and NCCN guidelines and are considered substandard in many European countries according to ESMO guidelines. In this report, we compare the sensitivity and specificity of ctDNA surveillance with imaging, measurement of CEA levels, and imaging plus measurement of CEA levels as recommended by the NCCN guidelines.

## Methods

In this retrospective cohort study, we aimed to compare the sensitivity of a Clinical Laboratory Improvement Amendments–certified ctDNA assay for minimal residual disease (Signatera) with standard radiographic imaging and measurement of CEA levels in identifying early disease recurrence in patients with curatively resected stage I to IV colorectal cancer. This retrospective study was approved by and conducted under the institutional review board of the City of Hope National Comprehensive Cancer Center, Duarte, California, which did not require informed consent for this retrospective outcome study. This study was conducted in accordance with the Strengthening the Reporting of Observational Studies in Epidemiology (STROBE) reporting guideline.

All patients with colorectal cancer undergoing surveillance with the Signatera ctDNA assay during a 2-year period (September 1, 2019, to November 30, 2021) were identified. Patients were separated into groups with resected stage II to III disease and resected stage IV disease. All patients undergoing minimal residual disease surveillance had undergone definitive surgical treatment and completed adjuvant therapy, if indicated. Patients followed a standard surveillance strategy that included ctDNA every 3 months for 2 years and then every 6 months for 3 years. Measurement of CEA levels was performed at the same interval as the ctDNA assay. Imaging studies were performed within NCCN guidelines and included yearly CT scans for 5 years for low-risk stage II disease and every 6 months for 2 years and then every year for 3 years for high-risk stage II and III disease. Imaging studies were performed every 3 months for 2 years and then every 6 months for 3 years for resected stage IV disease.

Recurrences were categorized as ctDNA-, imaging-, and CEA-detected recurrences. A ctDNA-detected recurrence was defined as any positive assay finding more than 4 weeks after definitive surgery. An imaging-detected recurrence was defined as any new metastatic lesion detected by CT or magnetic resonance imaging as reported by a board-certified clinical radiologist. A CEA-detected recurrence was defined as an abnormally elevated CEA level that was confirmed on 2 sequential reads. The sensitivity, specificity, and accuracy of each surveillance modality were measured in the context of the first sign of a confirmed recurrence. Confirmed recurrences included (1) any ctDNA-detected recurrence, given the recognized specificity of this assay; (2) an imaging-detected recurrence confirmed by biopsy findings or supported by tumor dynamics such as response to chemotherapy or progressive enlargement; and (3) a CEA level elevation that was subsequently confirmed by imaging, ctDNA, or histologic findings. Time from completion of definitive surgery to disease recurrence was calculated for each surveillance modality. The median time to recurrence was compared across all surveillance strategies. Data collection included patient demographics, tumor location, pathological and clinical stage of disease, site of resected metastatic disease in stage IV disease, radiology reports, pathology reports, CEA results, ctDNA results, and subsequent surgical interventions.

### Statistical Analysis

We estimated the sensitivity, specificity, positive predictive value (PPV), and negative predictive value (NPV) of ctDNA, imaging, measurement of CEA levels, and imaging plus measurement of CEA levels in being the first assay to detect the confirmed disease recurrence as identified in the Methods section.^16^ Calculations were performed separately for patients with stage II to III disease, patients with resected stage IV disease, and the overall population (stage II-IV). We used the McNemar χ^2^ test to determine the statistical significance of sensitivity and specificity of each modality for the overall population.

Kaplan-Meier curves were used to determine the median time to first sign of recurrence for all surveillance modalities. Two-sided *P* values (level of significance, *P* < .05) were computed using the log-rank test with GraphPad Prism software, version 7.04 (GraphPad).

## Results

### Patient Demographics

Patient demographics are detailed in Table 1. During the period of 2019 through 2021, 87 patients with a diagnosis of colorectal cancer underwent surveillance using the Signatera ctDNA assay. Only 48 patients with curative resections (28 men [58.3%] and 20 women [41.7%]; median age, 60 [IQR, 34-85] years; 31 [64.6%] with stage II-III and 17 [35.4%] with stage IV cancer) satisfied the inclusion criteria for this study and underwent surveillance by ctDNA assay, imaging, and measurement of CEA levels from September 1, 2019, to November 30, 2021 (eFigure 1 in the Supplement). Race data were collected from the electronic medical record; 10 patients (20.8%) were Asian; 1 (2.1%), Black; 34 (70.8%), White; and 3 (6.3%), not available. No therapeutic interventions were applied in all 48 patients during their surveillance. Thirty-two patients (66.7%) had received adjuvant therapy as part of their definitive therapy.

**Table 1.** Baseline Characteristics of Patients With Resected Colorectal Cancer

Characteristic	Patients (N = 48)^a^
Cancer stage	
II	15 (31)
III	16 (33)
IV^b^	
Overall	17 (35)
Resected liver	11 (23)
Resected lung	1 (2)
Resected other	4 (8)
MSS	41 (85)
MSI	4 (8)
Unknown	3 (6)
Left colon and rectum^c^	32 (67)
Right colon and transverse^c^	15 (31)
Race	
Asian	10 (21)
Black	1 (2)
White	34 (71)
Not available	3 (6)
Sex	
Men	28 (58)
Women	20 (42)
Age, median (range), y	60 (34-85)
Prior adjuvant chemotherapy	
Overall	32 (67)
Stage II	6 (12)
Stage III	12 (25)
Stage IV	14 (29)
Prior radiotherapy	
Overall	16 (33)
Stage II	3 (6)
Stage III	5 (10)
Stage IV	8 (17)

Abbreviations: MSI, microsatellite instability; MSS, microsatellite stability.

^a^ Unless otherwise indicated, data are expressed as the number (%) of patients. Percentages are rounded and therefore may not total 100.

^b^ One participant with resected liver metastases also had resection of a lung metastasis and was not included.

^c^ One participant had both left- and right-sided primary tumors and was not included.

### Disease Recurrence During Surveillance

Fifteen patients were confirmed to have recurrence as defined by the study (ctDNA positivity, imaging-identified recurrence confirmed by pathological findings or measurable disease dynamics). Twelve of the 15 patients with recurrence (80.0%) had prior adjuvant therapy; this was completed at a median of 4.7 (IQR, 1.4-11.0) months before recurrence. The details of prior therapy and the results of each of the surveillance assays in the 15 patients with recurrence are summarized in Table 2. Among the 15 patients with confirmed recurrence, ctDNA and imaging studies identified the recurrence concurrently in 3 patients (20%), only 1 of whom had elevated CEA levels at the time of confirmed recurrence. Seven of 15 patients (46.7%) had their confirmed recurrence identified by imaging despite a concurrent negative ctDNA finding, which was performed within 2 weeks of imaging recurrence. Five of these 7 patients (71.4%) had low-volume lung disease (eFigure 2 in the Supplement), 1 (14.3%) had a low-volume metastatic liver disease, and 1 (14.3%) had brain metastasis. Measurement of CEA levels exerted a low sensitivity in identifying disease recurrence, with only 3 patients (20.0%) having elevated CEA levels at the first sign of confirmed relapse: 1 patient with metastatic disease in the lung only was identified first by CEA levels and subsequently by imaging and pathological confirmation, with persistently negative ctDNA findings; 1 patient with metastatic disease involving multiple organs had concurrent imaging and ctDNA findings of recurrence; and 1 patient had concurrent ctDNA positivity and subsequent radiographic confirmation. When combining imaging plus measurement of CEA levels as a surveillance modality, 4 of 15 patients with recurrence (26.7%) had concurrent ctDNA positivity, whereas imaging plus measurement of CEA levels identified 7 patients (46.7%) with recurrence who had negative ctDNA findings at the time of recurrence (eFigure 2 in the Supplement). The ctDNA assay identified recurrence before imaging in 5 patients: 2 patients with solitary small metastatic disease in the liver, 1 patient with metastatic disease in the lung, and 2 patients with multiple distant lymph nodes metastases. Imaging recurrences in these 5 patients occurred at 1.1, 5.8, 6.1, 17.5, and 20.3 months after the finding of ctDNA positivity.

**Table 2.** Detailed Surveillance Information for Each Patient

Patient No.	Cancer stage	Time from definitive surgery, mo	Site of recurrence	Surveillance strategy by recurrence detection (order of detection)			
				ctDNA	Imaging	CEA levels	CEA levels plus imaging
1	II	>6	Lung	No	Yes (second)	Yes (first)	Yes (first)
2	II	<3^a^	Liver	Yes (first)	Yes (third)	Yes (second)	Yes (second)
3	II	>6	Lung	No	Yes (first)	No	Yes (first)
4	III	>6	RPLN	Yes (first)	Yes (second)	No	Yes (second)
5	III	>6	RPLN, mesenteric lymph node, and multiple other (supraclavicular, subclavicular, and axillary) lymph nodes	Yes (first)	Yes (second)	Yes (first)	Yes (first)
6	III	>6	Liver, lung, peritoneal, rectum	Yes (first)	Yes (first)	No	Yes (first)
7	IV	>6	Liver, RPLN, anastomotic recurrence, pelvic lymphadenopathy	Yes (first)	Yes (first)	No	Yes (first)
8	IV	>6	Liver	Yes (first)	Yes (second)	No	Yes (second)
9	IV	3-6^a^	Liver	Yes (second)	Yes (first)	No	Yes (first)
10	IV	>6	Lung	No	Yes (first)	Yes (second)	Yes (first)
11	IV	>6	Lung	Yes (first)	Yes (second)	No	Yes (second)
12	IV	>6	Lung	No	Yes (first)	No	Yes (first)
13	IV	>6	Liver, lung, RPLN	Yes (first)	Yes (first)	Yes (first)	Yes (first)
14	IV	>6	Cerebellum	No	Yes (first)	No	Yes (first)
15	IV	>6	Lung	No	Yes (first)	No	Yes (first)

Abbreviations: CEA, carcinoembryonic antigen; ctDNA, circulating tumor DNA; RPLN, retroperitoneal lymphadenopathy.

^a^ No adjuvant therapy; time of recurrence estimated from surgery on primary tumor in case of stages II to III and primary tumor resection or resection of metastases, whichever occur last, in case of stage IV disease.

The time to disease recurrence was estimated for each of the ctDNA, imaging, and imaging plus CEA modalities. No statistical difference was noted among all 3 modalities in the time to identifying a true recurrence (median 14.3 months for ctDNA vs 15.0 months for imaging vs 15.0 months for imaging plus CEA measurement) (Figure, A and B).

**Figure.**
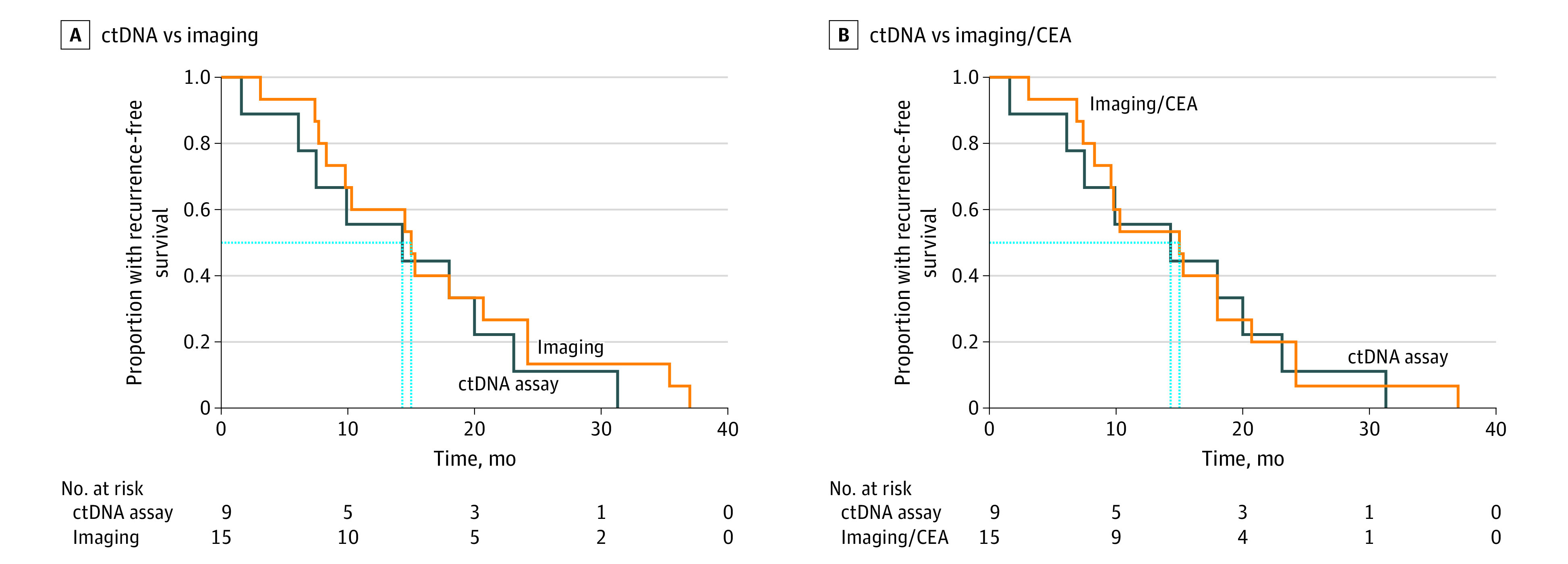
Recurrence-Free Survival in Patients With Resected Colorectal Cancer Surveillance strategies that were compared include a circulating tumor DNA (ctDNA) assay (Signetera; Natera), imaging, and imaging combined with measurement of carcinoembryonic antigen (imaging/CEA) levels. (A) ctDNA vs imaging, *P* = .45. (B) ctDNA vs imaging/CEA, *P* = .79. Dashed blue lines indicate the median recurrence-free survival for each modality.

### Sensitivity, Specificity, PPV, and NPV

The sensitivity, specificity, PPV, and NPV of ctDNA, imaging, and measurement of CEA levels were calculated based on the performance of each of the surveillance modalities in comparison with the confirmed recurrence as defined by the study (Table 3). Surveillance with CEA measurement appeared to perform poorly in detecting a first recurrence, with a sensitivity of only 20.0% (95% CI, 5.3%-48.6%). Circulating tumor DNA did not appear to perform numerically better than imaging, with sensitivities of 53.3% (95% CI, 27.4%-77.7%) and 60.0% (95% CI, 32.9%-82.5%), (*P* > .99), respectively. The specificity was the highest for ctDNA at 100% (95% CI, 87.0%-100%), which is expected because we defined any ctDNA-detected recurrence as a confirmed recurrence irrespective of subsequent imaging confirmation. The specificity of imaging was high at 96.9% (95% CI, 82.5%-99.8%), with 1 patient with low-risk stage III disease developing several suspicious lung nodules that were biopsied and confirmed to be benign (granulomatous) and therefore ruling out recurrence. When combining imaging and measurement of CEA levels, as recommended by NCCN guidelines, the combination modality had a numerical advantage compared with ctDNA in identifying a recurrence (sensitivity, 73.3% [95% CI, 44.8%-91.1%]; *P* = .55) and performed well on both the PPV (73.3% [95% CI, 44.8%-91.1%] vs 100% [95% CI, 59.8%-100%]) and NPV (87.9% [95% CI, 70.9%-96.0%] vs 82.5% [95% CI, 66.6%-92.1%]). Our statistical analysis showed that the sensitivity of CEA surveillance was significantly worse than that of combined imaging and measurement of CEA levels (20.0% [95% CI, 5.3%-48.6%]; *P* = .01). No other statistical difference in sensitivity and specificity was observed among surveillance modalities (Table 4).

**Table 3.** Sensitivity, Specificity, PPV, and NPV for ctDNA, Imaging, and CEA

Measure	Detection method, % (95% CI)			
	ctDNA	Imaging	CEA level	Imaging plus CEA level
Sensitivity	53.3 (27.4-77.7)	60.0 (32.9-82.5)	20.0 (5.3-48.6)	73.3 (44.8-91.1)
Specificity	100 (87.0-100)	96.9 (82.5-99.8)	90.9 (74.5-97.6)	87.9 (70.9-96.0)
PPV	100 (59.8-100)	90.0 (54.1-99.5)	50.0 (13.9-86.1)	73.3 (44.8-91.1)
NPV	82.5 (66.6-92.1)	84.2 (68.1-93.4)	71.4 (55.2-83.8)	87.9 (70.9-96.0)

Abbreviations: CEA, carcinoembryonic antigen; ctDNA, circulating tumor DNA; NPV, negative predictive value; PPV, positive predictive value.

**Table 4.** Statistical Comparison of Sensitivity and Specificity Among Surveillance Modalities in Overall Population

Surveillance modality	*P* value^a^	
	Sensitivity	Specificity
ctDNA vs imaging	>.99	>.99
ctDNA vs imaging plus CEA level	.55	.13
ctDNA vs CEA level	.13	.25
CEA level vs imaging	.11	.62
CEA level vs imaging plus CEA level	.01	>.99
Imaging vs imaging plus CEA level	.48	.25

Abbreviations: CEA, carcinoembryonic antigen; ctDNA, circulating tumor DNA.

^a^ Specificity and sensitivity measures by surveillance modality appear in Table 3.

### Surveillance Strategies and Association With Curative Interventions

Among the 15 patients with recurrences, 5 (33.3%) underwent interventions with curative intent. Two patients had their first evidence of relapse based on ctDNA findings and were subsequently found to have evidence of radiographic recurrence with a solitary lesion in the liver by positron emission tomography or CT and magnetic resonance imaging at 1.1 and 6.1 months after the positive ctDNA finding. The patient with positive findings on positron emission tomography underwent successful ablation, whereas the second patient underwent laparoscopic segmental resection of liver metastasis. Three patients identified with recurrence on CT in the lungs with a negative ctDNA assay finding underwent treatment with curative intent. Two patients underwent wedge resection using video-assisted thoracoscopic surgery and remain without evidence of recurrence, and the third patient underwent stereotactic body radiotherapy to his lung recurrence. All 3 patients continued to have negative ctDNA findings and remain recurrence free after being followed up for 4, 4, and 15 months.

## Discussion

The sensitivity of personalized ctDNA assays has drawn significant interest among medical communities. Many oncologists have opted to use ctDNA in the surveillance of early-stage and resected stage IV colorectal cancer. Much of the supporting evidence comes from a prospective clinical trial that showed a strong prognostic role for ctDNA in the follow-up of stage I to III colorectal cancer in a large Danish trial.^11^ However, this study was limited by the inadequate radiographic surveillance, which may have biased the overall outcomes to support the superiority of ctDNA over imaging in monitoring for recurrence. Another study^13^ suggested improved sensitivity of a more limited ctDNA platform over imaging, but again used infrequent imaging (yearly for 3 years) in a high-risk population.

In this study of 48 patients with stage II to IV colorectal cancer who were in a clinical remission after surgical intervention, we reported on the sensitivity and specificity of ctDNA, measurement of CEA levels, imaging, or imaging plus measurement of CEA levels (eTable in the Supplement). We were not able to confirm any advantages of the ctDNA assay over imaging in detecting disease recurrence. In fact, imaging plus measurement of CEA levels identified the recurrence before ctDNA in 7 of 15 cases and concurrently with ctDNA in 4 of 15 cases. Therefore, the sensitivity of the current NCCN guidelines in identifying disease recurrence appeared numerically superior to that of ctDNA. Computed tomographic imaging was particularly more sensitive in identifying pulmonary recurrence than ctDNA, with 5 of 8 patients with lung recurrences identified by CT before ctDNA or with persistently negative ctDNA findings. None of our patients with recurrent disease had peritoneal recurrence only. Therefore, the merits of ctDNA in patients with peritoneal recurrence could not be assessed in our study. However, prior studies have reported that patients with peritoneal disease have the lowest rate of detectable ctDNA, especially when compared with patients with liver and lung disease, raising concerns regarding the dependence on ctDNA in identifying such recurrences.^17,18^

Although ctDNA assay identified 5 patients before imaging, it is unlikely that this intervention would have changed the treatment of these patients. One patient was subsequently identified to have multiple lung metastases that were inoperable. Two patients underwent curative intent surgery after ctDNA but would have arguably experienced a similar intervention if followed up by standard surveillance. One of these 2 patients had an elevated CEA level after a positive ctDNA finding and before radiographic recurrence, which could have arguably triggered a positron emission tomography or CT as per NCCN considerations, therefore identifying the recurrence. The other patient had previously resected metastatic disease of the liver and was scheduled to undergo a second magnetic resonance imaging study in another 3 months after ctDNA positivity, which would have likely identified the recurrent lesion without compromising his clinical outcome. In addition, ctDNA identified 2 recurrences with persistently rising ctDNA levels for more than 1 year in 2 patients who eventually had evidence of disease recurrence at 17.5 and 20.3 months after ctDNA positivity (eFigure 3 in the Supplement). Both late radiographic recurrences had diffuse retroperitoneal disease recurrence and therefore were not candidates for curative-intent interventions.

Our study highlights the limitations of ctDNA in the surveillance of resected colorectal cancer and calls for additional studies before the universal adoption of ctDNA in clinical practice. In addition, our findings confirm the ongoing relevance of CT imaging in the follow-up of patients with resected colorectal cancer. More important, our findings bring into questions the reliability of the ctDNA assay in conferring a sense of security regarding the risk of disease recurrence in colorectal cancer. Although a positive ctDNA finding without doubt indicates an almost definitive risk of relapse, we show that a negative ctDNA finding is common in the setting of low-volume metastatic disease, especially in metastatic disease of the lung. Numerous phase 3 clinical trials in stage II and III colorectal cancer are currently evaluating a dose de-escalation or the elimination of adjuvant chemotherapy in patients with negative ctDNA findings.^19,20,21^ In the absence of large observational studies to vet the sensitivity and predictive value of negative postoperative ctDNA findings in well-characterized populations with stage III disease (stratified by T category, N category, location, grade, lymphovascular invasion, and molecular and immune characteristics), those clinicians may have taken a giant leap of faith by endorsing ctDNA assays as predictive and prognostic. The BESPOKE study is evaluating the impact of the Signatera ctDNA assay on decision-making and identification of colorectal cancer disease relapse among patients undergoing surgery for metastases through a large 2000-participant case-control study.^22^ However, the case-control design and the lack of standardization of imaging surveillance intervals across centers will limit the interpretation of the results.

### Limitations

Our study is limited by its small size, small number of recurrences, and short follow-up. We defined ctDNA-detected recurrence as a true recurrence despite the lack of a mandate of a radiographic progression; however, our decision to proceed with such a definition was based on supporting evidence that persistent ctDNA positivity has been associated with recurrence in all patients in 2 studies.^11,12^ In addition, delayed recurrences have been seen in patients with colorectal cancer, although infrequently.^23^ Hence, the lack of radiographic recurrence, even at 5 years, in a patient with persistent ctDNA positivity does not preclude the presence of minimal residual disease or later recurrences. Nonetheless, all our patients with ctDNA-detected relapses did eventually experience radiographic recurrence and hence represent true-positive findings by radiographic criteria, adding further strength to our findings. Our statistical analysis did not show any significant difference in sensitivity and specificity between measurement of CEA levels and ctDNA, as well as combined measurement of CEA levels and imaging, probably reflecting that our analysis is underpowered by the small sample size. Additional limitations include the heterogenous patient population by stage and treatment, which may limit the generalization of our data within different populations with colorectal cancer. Nonetheless, the correlation between low-burden lung recurrence and negative ctDNA findings is striking and calls for larger prospective studies to assess the sensitivity of ctDNA vs standard of care surveillance. Such studies should be standardized to include intensive surveillance approaches as supported by NCCN and ESMO guidelines.

## Conclusions

The findings of this prospective cohort study suggest that ctDNA assay may not provide definitive advantages as a surveillance strategy compared with standard imaging combined with measurement of CEA levels when performed per NCCN guidelines. The correlation between low-burden lung recurrence and negative ctDNA findings should be investigated further in larger prospective studies.

## Notes

Supplement. **eFigure 1.** Study Flow Diagram **eFigure 2.** Images From Patients With Lung Recurrence With Negative ctDNA Findings **eFigure 3.** Images From Patients With Persistent ctDNA Positivity and With Delayed Radiographic Disease Recurrence **eTable.** Sensitivity, Specificity, PPV, and NPV for ctDNA, Imaging, and CEA Levels
