# Supplementary material for: Evaluation of Comparative Surveillance Strategies of Circulating Tumor DNA, Imaging, and Carcinoembryonic Antigen Levels in Patients With Resected Colorectal Cancer
Source: JAMA Netw Open. 2022 Mar 8;5(3):e221093. doi: 10.1001/jamanetworkopen.2022.1093 (PMC8905389; doi:10.1001/jamanetworkopen.2022.1093)
Supplement: Supplement. — eFigure 1. Study Flow Diagram eFigure 2. Images From Patients With Lung Recurrence With Negative ctDNA Findings eFigure 3. Images From Patients With Persistent ctDNA Positivity and With Delayed Radiographic Disease Recurrence eTable. Sensitivity, Specificity, PPV, and NPV for ctDNA, Imaging, and CEA Levels [file jamanetwopen-e221093-s001.pdf]

Supplementary Online Content

Fakih M, Sandhu J, Wang C, et al. Evaluation of comparative surveillance strategies of circulating tumor DNA, imaging, and carcinoembryonic antigen levels in patients with resected colorectal cancer. *JAMA Netw Open*. 2022;5(3):e221093. doi:10.1001/jamanetworkopen.2022.1093

eFigure 1. Study Flow Diagram

eFigure 2. Images From Patients With Lung Recurrence With Negative ctDNA Findings

eFigure 3. Images From Patients With Persistent ctDNA Positivity and With Delayed Radiographic Disease Recurrence

eTable. Sensitivity, Specificity, PPV, and NPV for ctDNA, Imaging, and CEA Levels

This supplementary material has been provided by the authors to give readers additional information about their work.

© 2022 Fakih M et al. *JAMA Network Open*.

eFigure 1. Study Flow Diagram

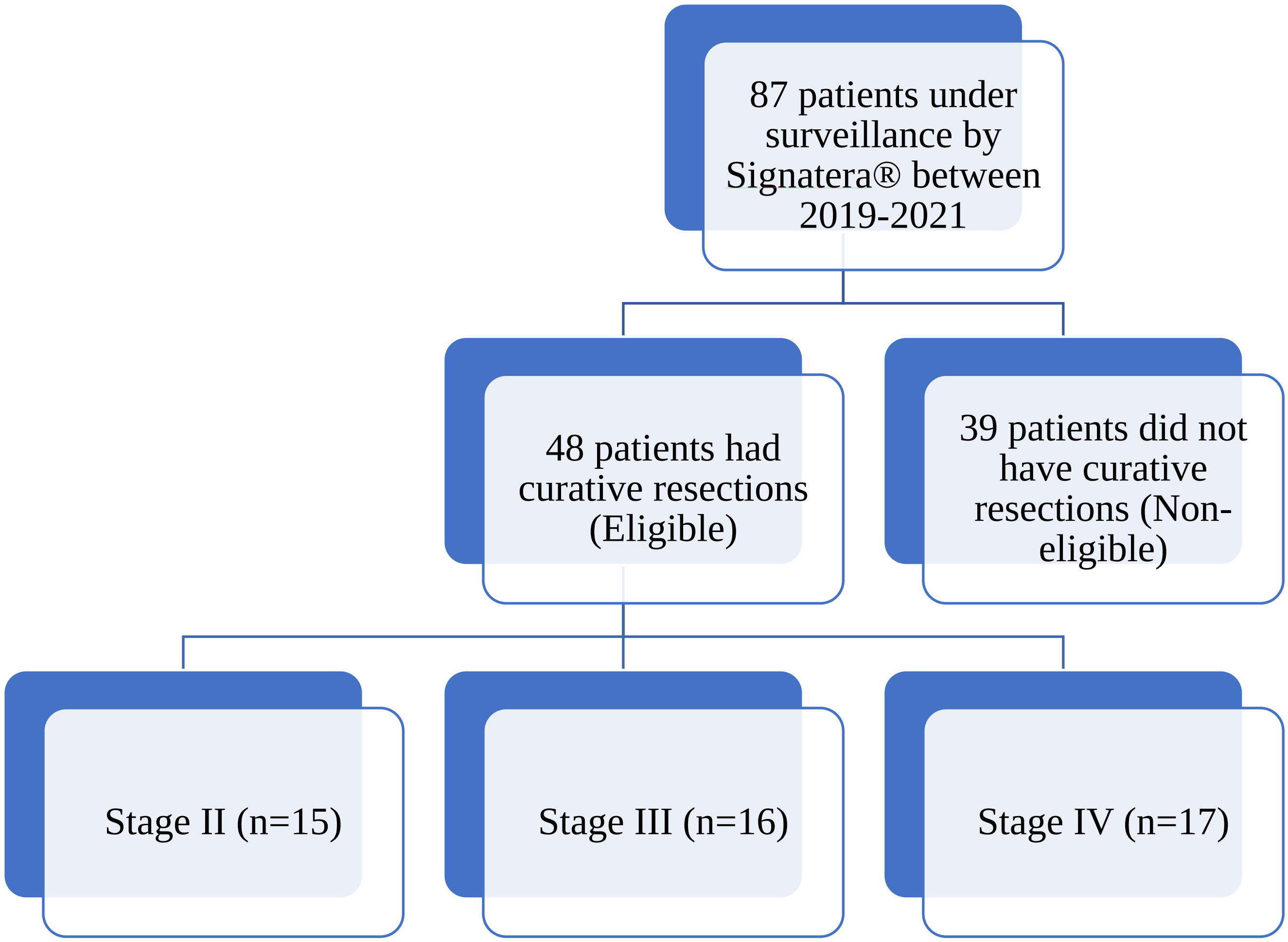

© 2022 Fakih M et al. *JAMA Network Open*.

eFigure 2. Images From Patients With Lung Recurrence With Negative ctDNA Findings

Case 1: A medial left upper lobe lesion that was subsequently resected confirming metastatic colorectal cancer

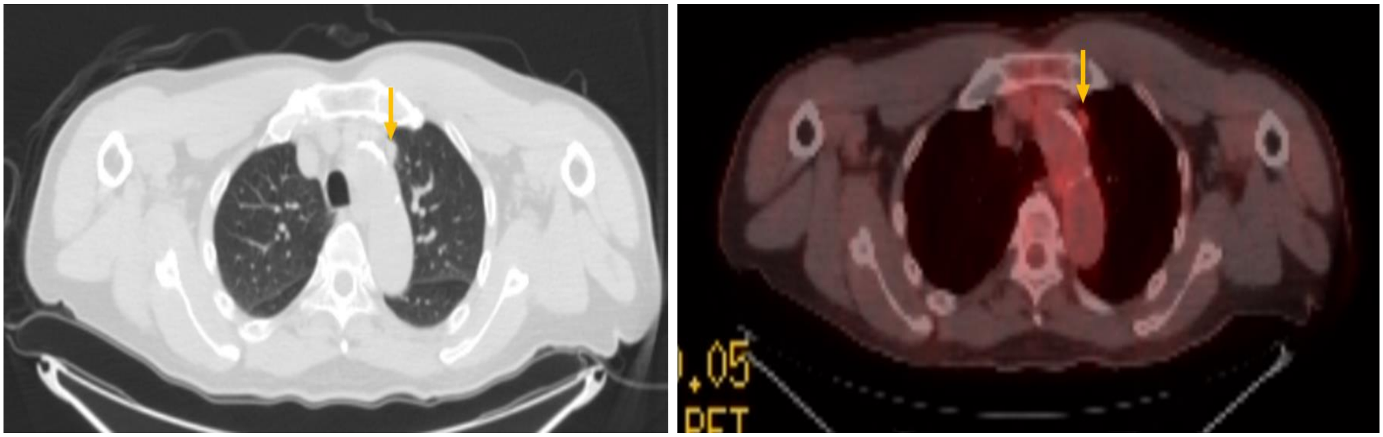

Case 2: Multiple lung nodules that one of which (included in image) was subsequently resected confirming metastatic colorectal cancer

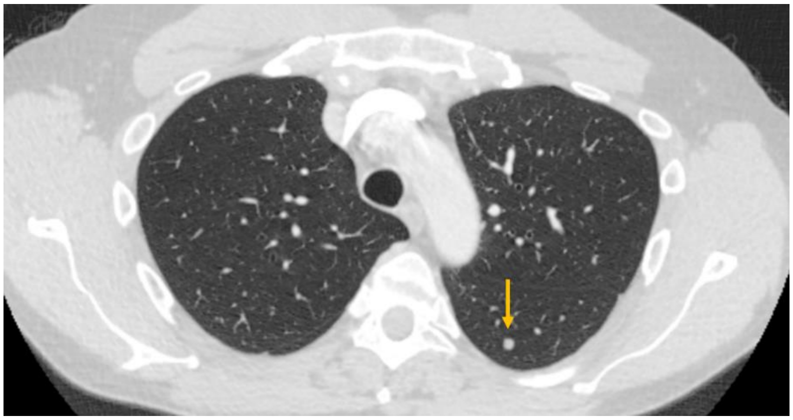

Case 3: Multiple lung nodules associated with a rise in CEA and subsequently resolved with chemotherapy

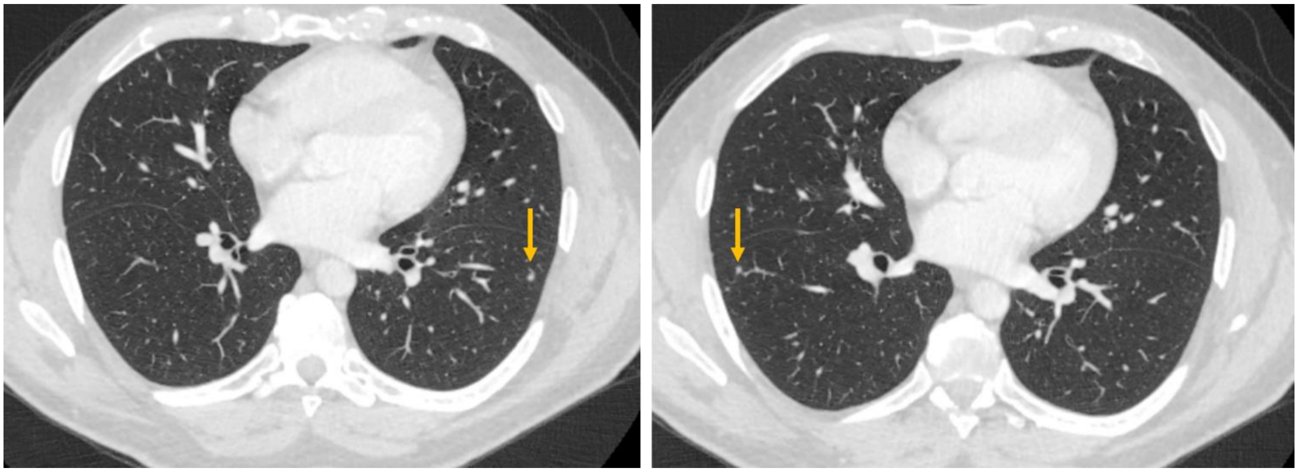

© 2022 Fakih M et al. *JAMA Network Open*.

Case 4: Enlarging left lower lobe nodule over a period of 4 months. The lesion was treated with curative intent with stereotactic body radiation therapy

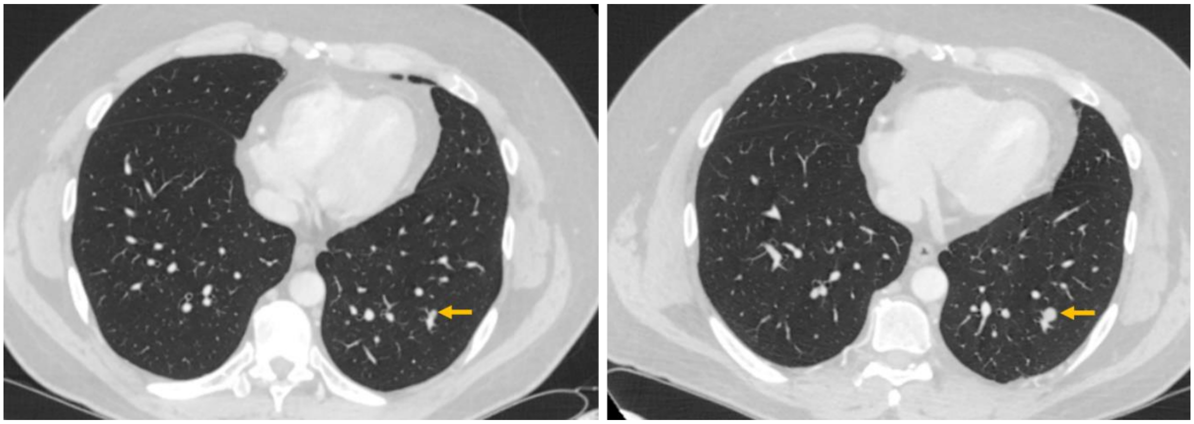

Case 5: Enlarging pleural base right upper lobe nodule over a period of 4 months. Resection confirmed metastatic colorectal cancer

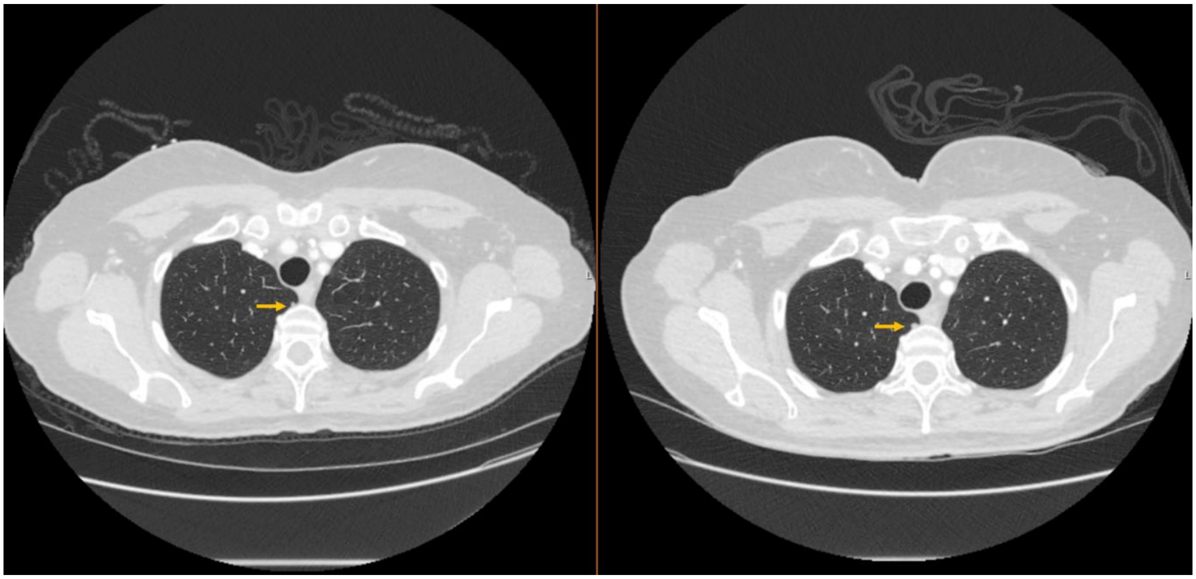

© 2022 Fakih M et al. *JAMA Network Open*.

eFigure 3. Images From Patients With Persistent ctDNA Positivity and With Delayed Radiographic Disease Recurrence

Case 1: Increase in one of several retroperitoneal lymph nodes (blue arrow) confirmed on CT (right) compared to 6 months prior (left)

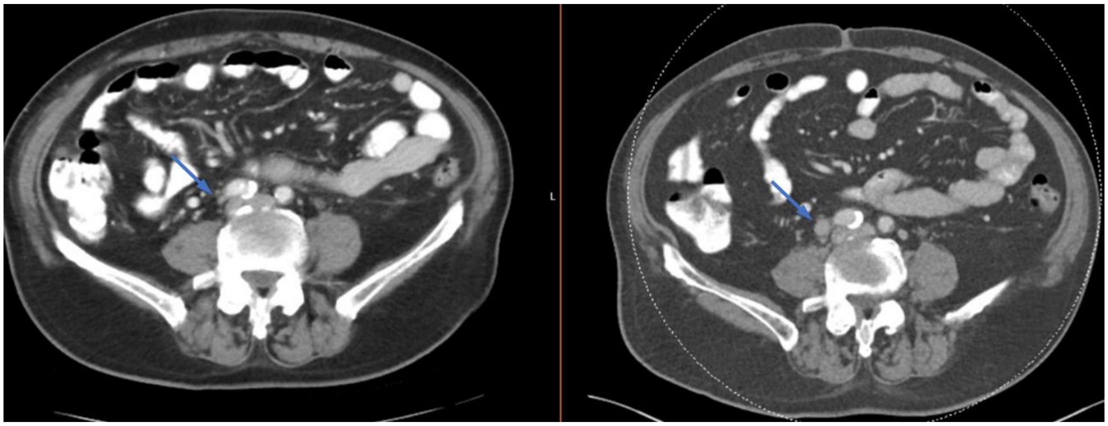

Case 2**:** Multiple newly FDG positive lymph nodes (blue arrow) noted on PET/CT

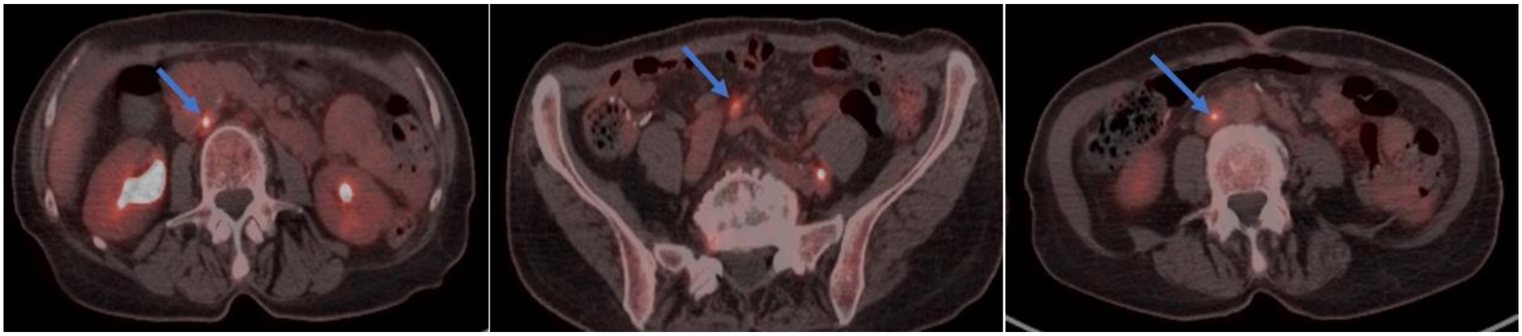

© 2022 Fakih M et al. *JAMA Network Open*.

eTable. Sensitivity, Specificity, PPV, and NPV for ctDNA, Imaging, and CEA Levels

	ctDNA %, (95% CI)			Imaging %, (95% CI)			CEA %, (95% CI)			Imaging or CEA %, (95% CI)		
Stage	II-III	IV	II-IV	II-III	IV	II-IV	II-III	IV	II-IV	II-III	IV	II-IV
Sensitivity	66.7, (24.1-94)	44.4, (15.3-77.3)	53.3, (27.4-77.7)	33.3, (6-75.9)	77.8, (40.2-96.1)	60, (32.9-82.5)	33.3, (6-75.9)	11.1, (0.6-49.3)	20.0, (5.3-48.6)	66.7, (24.1-94)	77.8, (40.2-96.1)	73.3, (44.8-91.1_
Specificity	100, (83.4-100)	100, (59.8, 100)	100, (87-100)	96, (77.7-99.8)	100, (59.8-100)	96.9, (82.5-99.8)	88, (67.7-96.8)	100, (59.8-100)	90.9, (74.5-97.6)	84, (63.1-94.7)	100, (59.8-100)	87.9, (70.9-96)
PPV	100, (39.6-100)	100, (39.6, 100)	100, (59.8-100)	66.7, (12.5-98.2)	100, (56.1-100)	90, (54.1-99.5)	40, (7-83)	100, (5.5-100)	50, (13.9-86.1)	55.6, (17.4-82.6)	100, (56.1-100)	73.3, (44.8-91.1)
NPV	92.6, (74.2-98.7)	61.5, (32.3-84.9)	82.5, (66.6-92.1)	85.7, (66.4-95.3)	80, (44.2-96.5)	84.2, (68.1-93.4)	84.6, (64.3-95)	50, (25.5-74.5)	71.4, (55.2-83.8)	91.3, (70.5-98.5)	80, (44.2-96.5)	87.9, (70.9-96)

© 2022 Fakih M et al. *JAMA Network Open*.
